# ISMAC: An Intelligent System for Customized Clinical Case Management and Analysis

**DOI:** 10.1155/2015/473168

**Published:** 2015-10-01

**Authors:** Mingyu You, Chong Chen, Guo-Zheng Li, Shi-Xing Yan, Sheng Sun, Xue-Qiang Zeng, Qing-Ce Zhao, Liao-Yu Xu, Su-Ying Huang

**Affiliations:** ^1^Department of Control Science and Engineering, Tongji University, Shanghai 200000, China; ^2^Computer Center, Nanchang University, Nanchang 330000, China; ^3^Shanghai Institute of Traditional Chinese Medical Literatures, Shanghai 200000, China

## Abstract

Clinical cases are primary and vital evidence for Traditional Chinese Medicine (TCM) clinical research. A great deal of medical knowledge is hidden in the clinical cases of the highly experienced TCM practitioner. With a deep Chinese culture background and years of clinical experience, an experienced TCM specialist usually has his or her unique clinical pattern and diagnosis idea. Preserving huge clinical cases of experienced TCM practitioners as well as exploring the inherent knowledge is then an important but arduous task. The novel system ISMAC (Intelligent System for Management and Analysis of Clinical Cases in TCM) is designed and implemented for customized management and intelligent analysis of TCM clinical data. Customized templates with standard and expert-standard symptoms, diseases, syndromes, and Chinese Medince Formula (CMF) are constructed in ISMAC, according to the clinical diagnosis and treatment characteristic of each TCM specialist. With these templates, clinical cases are archived in order to maintain their original characteristics. Varying data analysis and mining methods, grouped as Basic Analysis, Association Rule, Feature Reduction, Cluster, Pattern Classification, and Pattern Prediction, are implemented in the system. With a flexible dataset retrieval mechanism, ISMAC is a powerful and convenient system for clinical case analysis and clinical knowledge discovery.

## 1. Introduction

Traditional Chinese Medicine (TCM) is one of the main approaches for disease diagnosis and treatment in China [1, 2]. The basic theories were formed more than 2000 years ago [3], and according to the reported data of Chinese National Bureau of Statistics [4], there were 2688 TCM hospitals, receiving 8.89 million inpatients and 275 million outpatients in China 2008. It is widely accepted in China that TCM is safer and more efficient for some chronic and intractable illnesses. In past decades, TCM has been increasingly adopted around the world as the complementary medical therapy for various diseases such as cancer [5], rheumatoid arthritis [6], leukemia [7], H1N1 Virus [8], and migraines [9]. In TCM diagnosis, discomforts in different parts of the body are taken into consideration together to find the root pathological cause. TCM also maintains that the health of individual human beings is intimately involved with the environment. Excellent TCM practitioners pursue the discovery of potential disease before it may be perceived by the patient or examined by medical instruments. The master goal of TCM is preventative: a common modern desire.

As opposed to modern biomedical science, diagnostic knowledge and TCM herb formulas are mostly developed through practice. Veteran TCM practitioners usually have more than 30 years of clinical experience. Moreover, all TCM experts have a unique understanding of TCM philosophy, which results in unique diagnosis and treatment patterns. Therefore, to archive clinical data as well as maintain different characteristics in one system is a difficult but vital task in TCM informatics.

TCM clinical cases archive the important experience of veteran TCM practitioners and store useful information for young TCM physicians. The analysis of clinical data can help in the extraction of vital clinical strategies for TCM knowledge accumulation and promotion [10]. Clinical records are important for the understanding of theory and intellectual gain for new doctors. In a traditional sense, successors follow their teachers and study the in-depth knowledge while developing personal ability. But nowadays, with the unprecedented growth of clinical data, it is difficult to extract new knowledge from the data mountain. Data mining is a distinguished method of tracking underlying information. A great deal of research is dedicated to TCM data mining [11–13]. This indicates a promising future for TCM data mining, but most research contributes to TCM literature mining, herb knowledge modeling, gene network analysis, and the role of the understanding of herb components. None of this focuses on TCM clinical case analysis, most especially clinical records from highly experienced TCM practitioners.

A powerful, intelligent system is key for clinical case collection and analysis. Tian et al. [14] propose a platform for medical image processing and analyzing. It is powerful and useful but is designed specifically for medical image processing, which is a different task from clinical data management. Masseroli and Marchente [15] and Ku and Huang [16] propose efficient platforms for healthcare requirements, both of which are based on the web structure and provide convenient services to the patient. However, none of the platforms have been designed for TCM clinical cases. Chen et al. [17] demonstrate a grid-based TCM system, which also extends to a semantic-based database grid. Zhou et al. [18] developed TCMMDB, a unified, web-accessible multidatabase query system which has already integrated more than 50 databases, and CDW [11], a clinical data warehouse which incorporates the structured electronic medical record (SEMR) data for medical knowledge discovery and TCM clinical decision support (CDS). These systems provide platforms for TCM clinical data or herb collection but pay little attention to the clinical cases of high-experienced TCM practitioners. As opposed to strictly standardized modern biomedicine, the clinical cases of TCM practitioners, especially veteran practitioners, have obvious personal characteristics [19], which greatly distinguish many personal cases from others. The philosophy of syndrome differentiation from a patient's symptoms varies among different veteran practitioners. This variation may be the embodiment of the TCM veteran practitioner's thinking mode and knowledge system: more effort should be taken to preserve and analyze these systems. There are many tasks in the analysis of clinical records, such as symptom reduction, syndrome classification, and core formula mining. Since the data has the characteristics of being high dimension, multiclass, imbalance, the discovery of knowledge in clinical records is challenging.

In this paper, we present a novel system designed exactly for the customized management of TCM clinical cases and discuss the intelligent techniques for clinical data analysis and knowledge mining. The overview of our system has been proposed [20]. The rest of the paper is arranged as follows. An overview of this system is demonstrated in Section 2. The details for the design of the case management and analysis are introduced in Sections 3 and 4, respectively. An example for the system usage with clinical data from Mr. Zhang is provided in Section 5. Finally, Section 6 concludes the paper and presents a future perspective.

## 2. Platform Overview

**Figure 1:**
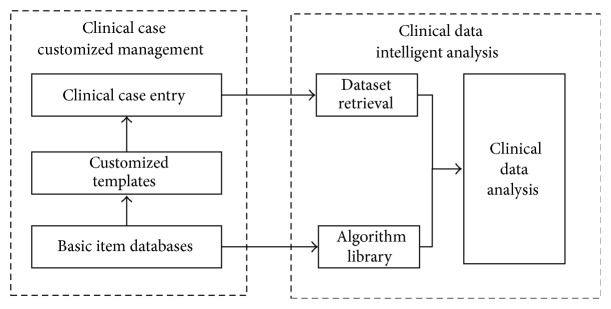
Functional overview of the platform.

Functional overview of ISMAC is illustrated in Figure 1. The customized management and intelligent analysis of TCM clinical cases are the two main parts in the system. The customized template is the critical element in data customized management. The template is defined as the entry format designed for clinical cases. It provides a predefined layout for specific doctor's medical records, which contain information like patient demographic information, symptoms, examinations, syndromes, and therapeutic principles. In ISMAC, we customize the entry template for each doctor, according to diagnosis methodology. The basic Item Database is set up to provide a foundation for the creation of the Customized Template, and Clinical Case Entry can be efficient and effective with the suitable Customized Template. As for intelligent analysis, algorithms and datasets are the two bases. ISMAC integrates not only popular algorithms in statistic analysis and data mining, but also some newly proposed methods in machine learning, in order to build a library for TCM clinical case intelligent analysis. In conjunction with the flexible Dataset Retrieval, the Algorithm Library makes various kinds of intelligent analysis possible in ISMAC.

**Figure 2:**
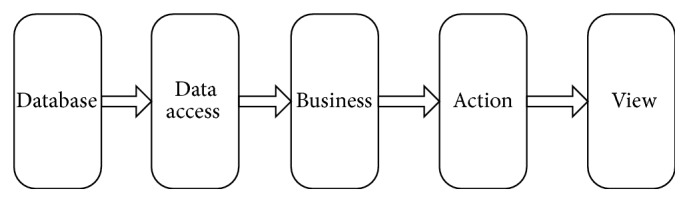
Framework of the platform.

ISMAC is implemented as a web-based application using the classical SSH (Struts + Spring + Hibernate) framework. As shown in Figure 2, it consists of 5 layers. The Database Layer contains tables storing various information, ranging from basic items such as symptom and syndrome to complex items, like knowledge models and learning algorithms. In the Data Access Layer, the Object-Relational Mapping (ORM) mechanism is used for convenient data manipulation. The top three layers, Business, Action, and View, are configured as the Model-View-Controller (MVC) pattern, in which the Business Layer manages the behavior and data in the application domain, the View Layer manages the display of different information, and the Action Layer interprets the mouse and keyboard inputs from the user and informs the Business Layer and the View Layer to react appropriately.

## 3. Custom Management of Clinical Case

The customized management of clinical cases is the core function in the ISMAC system, as archiving the clinical cases of different veteran TCM practitioners using the same entry form may submerge important knowledge in the clinical data.

### 3.1. Overall Design

**Figure 3:**
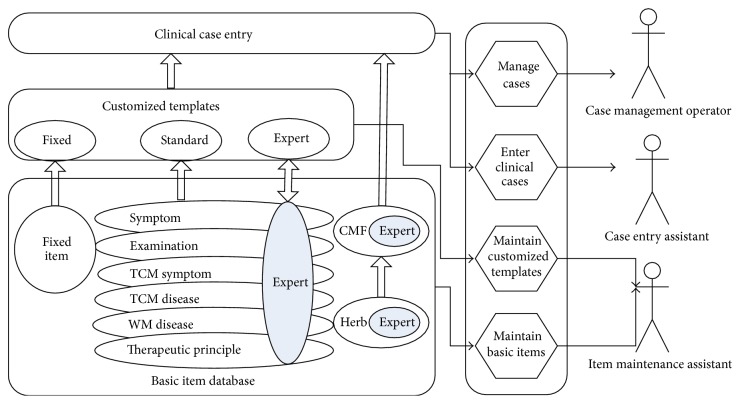
Overall design of the customized management of clinical case.

The overall design of the Customized Management of Clinical Case is shown in Figure 3, which illustrates the left part of Figure 1 in detail. Members of the Basic Item Database are listed. Varying libraries in the Basic Item Database possess the same structures but store different kinds of items: standard items and expert-standard items. A Customized Template consists of three parts: fixed, standard, and expert. These three parts are built up with different basic items. Clinical Case Entry depends on not only Customized Template but also the CMF and Herb item libraries.

Figure 3 also tells us that the task of customized management is divided into four subtasks, and charged by three roles. This is to increase work efficiency and protect private information. The three roles are Item Management Assistant (IMA), Case Entry Assistant (CEA), and the Case Management Operator (CMO), which is, respectively, assigned tasks of basic item maintenance and customized template maintenance, case input, and case management.

More details about the function of each part in customized management and its implementation technologies are introduced in the following.

### 3.2. Introduction of Functions

#### 3.2.1. Basic Item Database

Basic Item Database targets supporting template creation and case entry. As illustrated in Figure 3, libraries included in it can be classified into three categories for distinct usages: fixed part, standard part, and expert customized part. The last two categories may store two different kinds of items: standard items and expert-standard items.

The fixed Item Library in the left contains items such as patient name and created date. This is entitled “fixed” because these items appear in every template, and the library determines the fixed part of templates.

The Herb Library and CMF Library on the right store items about herbs and CMFs. Each CMF is composed of several herbs and corresponding dosages. The contents of these two libraries have two sources: one is* the Pharmacopoeia of the People's Republic of China* and the other contains each specialist's experience. Items from the first source are entitled standard items in ISMAC, and those from second source are tagged as expert-standard. In ISMAC, the two libraries directly serve Case Entry. CEAs choose items from this when filling a specific template.

The third category of item libraries is shown in the middle of the Basic Item Database. They work for the customization of templates. A customized itemset for a specific disease or syndrome is picked up from these libraries, to constitute a template together with fixed items. Six libraries are embodied in this category, including the Symptom Library, Examination Library, TCM Syndrome Library, TCM Disease Library, WM Disease Library, and Therapeutic Principle Library, as they are normal parts of a TCM clinical case. Similarly, both Herb and CMF libraries have standard and expert-standard items. The standard part comes from national or sectorial standards. The expert-standard part is the summary of veteran TCM practitioners experience.

#### 3.2.2. Customized Templates

Customized Templates are the core and basis for clinical case customized management.

The characteristics of TCM clinical cases prompt us to propose customized templates for a particular specialist. There are thousands of items carried in TCM diagnosis. However, almost all of the TCM practitioners take a subset of these items into consideration in clinical practice. Moreover, with different clinical experience and personal understanding of TCM theory, each practitioner has a different approach to diagnosis and clinical explanation. Providing a uniform sheet for data entry would cause two significant problems: clinical case entry would be inefficient (CEAs have to go through many unused items), and a uniform sheet cannot retain the clinical thinking of veteran TCM doctors in original taste. The knowledge of the TCM clinic is vital to TCM theory development and therefore deserves more attention and protection.

Three categories of basic items are used in Customized Template creation. Fixed items will be added to a template automatically. Standard items can be easily chosen from corresponding libraries in the Basic Item Database. For expert-standard items, those previously saved in the database can be selected like standard items. Otherwise, manual entry in a matched box is needed.

Customized Templates contribute largely to ISMAC's customization feature. However, this does not weaken its standardization. Most items in templates are standard or expert-standard items from the database. Items possessing the same names and IDs must refer to the same database. Multi-items for the same symptom or syndrome are forbidden. Such mechanisms ensure customization while retaining standardization.

#### 3.2.3. Clinical Case Collection and Management

With the establishment of Customized Templates and Basic Item Database, ISMAC will autogenerate a form with corresponding textboxes or checkboxes for the CEA to type in cases. A case usually contains the basic information of the patient and his diagnosis information, such as symptoms, syndromes, and CMF in every clinical visit. In some other systems, diagnoses resulting from different visits are usually treated as different clinical cases, which should repeat basic information entry and cut the strong relation between clinical visits. ISMAC links information of all visits together. With accumulated entry cases, more advanced data management operations can be created. We maintain that case management can be executed only by a CMO, who is often a doctor. Usually, he can check how many diagnoses he has conducted and review some important ones. He is also responsible for the verification of cases. Moreover, he can export his case records for backup, or other purposes.

#### 3.2.4. Access Control

As mentioned above, four tasks are assigned to three roles in ISMAC. Technically, the function is realized by the system's access control mechanism, which includes both role-based permission assignment and specialist-specific data access.

The access control is set up mainly for information security, which is important to the field of medicine and health care. Patients usually do not want clinical data viewed by others, except for physicians. On the other hand, the division of labor increases work productivity.

Role-based permission assignment in customized management part involves IMA, CMO, and CEA. IMA is created as an individual role, taking charge of maintenance (creation, update, etc.) for basic items and templates. They help build up the system but have no permission to input or read any clinical cases. DMO, always the physician, can check their own cases and execute jobs like case verification and export. The CEA helps enter the clinical cases and has no access to any stored data.

Specialist-specific data access is intended to limit all operators to access data, except for the appropriate physician. This means that a physician can only view their own clinical cases, and IMAs can only maintain items for a specific specialist, and CEAs can only enter cases with templates belonging to a particular physician.

## 4. Clinical Data Intelligent Analysis

Clinical case intelligent analysis is important for expert knowledge understanding and TCM theory development. The diagnosis methods and therapeutic means in TCM are the summation of clinical trials by intelligent specialists over many generations. However, in modern society, it is difficult and inefficient for physicians to learn TCM practical knowledge from solely clinical and personal experience. Intelligent analysis and advanced methods can provide a rapid investigation of clinical data. This knowledge and models may be difficult to retrieve with human intelligence.

### 4.1. Overview Design

**Figure 4:**
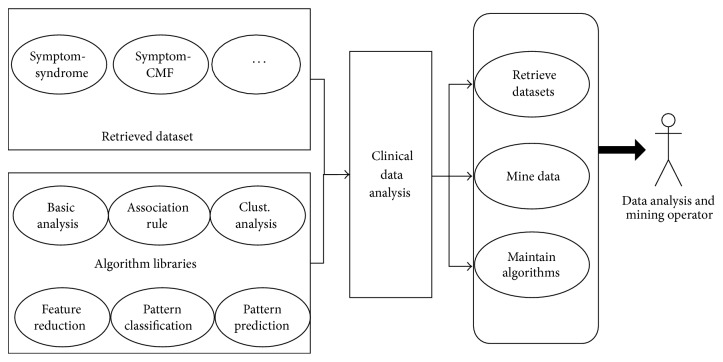
Overview design of intelligent data analysis.

Figure 4 details the right part of Figure 1. Dataset Retrieval is flexible, and various kinds of attribution combinations can be retrieved from the clinical case database. For example, datasets with symptom and syndrome attributes can be retrieved for the purpose of syndrome classification, while a dataset with CMF information can only be retrieved for core CMF mining. The Algorithm Library contains algorithms categorized into six groups: Basic Analysis, Association Rule, Feature Reduction, Cluster, Pattern Classification, and Pattern Prediction. With datasets and algorithms, varied intelligent analysis tasks for TCM clinical cases can then be conducted.

Similar to clinical case customized management, the work of intelligent analysis is also divided into several tasks. All the tasks are associated closely and assigned by the Data Analysis and Mining Operator (DAMO). Specialist-specific data access functions in access control mechanisms are also limiting. Each DAMO can only access the data (including cases and templates) of a specific TCM specialist.

### 4.2. Introduction of Functions

#### 4.2.1. Dataset Retrieval

**Figure 5:**
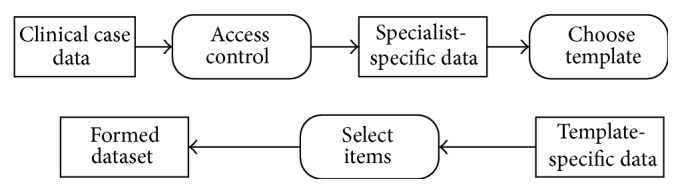
Data stream diagram of dataset retrieval.

The first step of data analysis is to retrieve the target dataset. A target dataset is the one that belongs to a target specialist and a certain template and contains necessary record items. The data stream diagram is shown in Figure 5. Because each DAMO is only capable of accessing one specialist, Access Control helps to ensure the correct choice of specialist. Then, the target template and items can be chosen through a corresponding user interface. And finally, the formed dataset is filtered out from the massive clinical case data. Information of each formed dataset is stored in ISMAC's database for multiple usage. It may be conveyed into  .arff files and used by* Weka*-based algorithms. Furthermore, this can also be exported in a  .csv format for sharing, or further investigation with other tools.

#### 4.2.2. Algorithm Library

The next step in data analysis is to choose the proper algorithms with suitable parameters. Many algorithms based on Weka are integrated in ISMAC.* Weka* [21] is one of the most popular toolkits in the field of machine learning and data mining, which is written in Java. To avoid reinventing the wheel, we wrap* Weka* into ISMAC. However, algorithms in* Weka* are not enough to deal with TCM clinical cases. Thus the extension of* Weka* has been made to include our newly proposed methods. All algorithms built in ISMAC are summarized as follows. 

*Algorithms Organized as Six Groups*

 Basic Analysis
   Statistics computing
   Visualization tools
 Feature Reduction
   PCA
   ICA
   CFS
   IG
   Im-IG
 Pattern Classification
   NaiveBayes
   SVM
   J48
   PLSC
   APLSC
   MAPLSC
 Association Rule
   Apriori
   AprioriTid
   Fp-tree
   AVM
 Cluster
   EM
   
*K*Means
 Pattern Prediction
   Linear Regression
   Logistic Regression.

*Training Section of MAPLSC*

 Training dataset *D*, the number of classes *k*
 MAPLSC classifier:
 (1) Set up *k*(*k* − 1)/2 subsets from the dataset *D*, each subset *S*_*ij*_ is composed of the examples from class *C*_*i*_ and class *C*_*j*_.
(2) Train classifier *APLSC*_*ij*_ with the examples in *S*_*ij*_, and obtain output *f*_*m*_ from *APLSC*_*ij*_ for each example *x*_*m*_ in *S*_*ij*_.
  (3) Calculate posterior probability parameters *A*_*ij*_ and *B*_*ij*_ with (1) and *f*_*m*_.
  (4) Output *MAPLSC* classifier with *k*(*k* − 1)/2  *APLSC*_*ij*_ and the corresponding *A*_*ij*_ and *B*_*ij*_.

*Testing Section of MAPLSC*

 MAPLSC classifier and corresponding posterior probability parameters, test sample *x*_*t*_, predicted label $\tilde{y_{t}}$ for test sample *x*_*t*_:
  (1) Calculate the output *f*_*t*_^*ij*^ from *APLSC*_*ij*_ for test example *x*_*t*_.
  (2) Calculate the posterior probability output *r*_*ij*_ = Prob(*C*_*i*_∣*x*_*t*_) by (1), with parameters *A*_*ij*_, *B*_*ij*_ and *f*_*t*_^*ij*^.
  (3) Combine the *k*(*k* − 1)/2  *r*_*ij*_ by (2), and get the probabilities on *k* classes $\left(\tilde{p_{1}},\tilde{p_{2}},\tilde{p_{3}},\tilde{p_{4}}\right)$.
  (4) Predict label for *x*_*t*_ by $\tilde{y_{t}}=arg{max}_{i}p_{i}$.

Basic Analysis Algorithm Group contains algorithms for basic statistical analysis and data visualization. The cluster algorithm is intended to cluster instances with similar properties. The Association Rule Library provides methods that help find frequent patterns and association rules from a data set.* Apriori* [22] is an important algorithm for frequent pattern mining, and many other methods are based upon it.

Feature Reduction helps to reduce the volume of the feature set yet maintains the majority of the original information. It can be divided into two branches, feature extraction and feature selection. Feature extraction applies data encoding or transformation to obtain the “compressed” presentation of original data, while feature selection tries to remove irrelevant, weakly relevant, and redundant features. One new feature of selection algorithms proposed by us is integrated in ISMAC, known as* Im-IG* [23], an improved Information Gain (IG) for imbalance (Im) problem.

Pattern Classification and Pattern Prediction algorithms are used to build classification and prediction models. Pattern classification assigns one or several categorical (discrete, unordered) labels to a given instance. Pattern Prediction algorithms assign input instances to continuous prediction values. Three of our proposed novel methods are provided in the Pattern Classification Algorithm Group. PRIFEAB adopts asymmetric bagging and feature selection to address the imbalanced problem.* APLSC* [24] and* MAPLSC* [25] also attempt to address the imbalance problem for binary and multiple problems, respectively, based on the Partial Least Squares Classifier (PLSC).

#### 4.2.3. Novel Method, MAPLSC

Here we would like to introduce in detail one of the novel methods, MAPLSC, which is integrated in ISMAC. MAPLSC is based on the APLSC algorithm. It combines multiple one-versus-one APLSC binary classifiers to solve the multiclass imbalance problem. The pseudocode of MAPLSC is illustrated in the previous section.

In the training section, one-versus-one binary classifiers for all classes are first trained with the APLSC algorithm. After that, (1) is used to transfer output *f* of APLSC to posterior possibility parameters *A* and *B*, used for classifier combinations:

(1) $$\begin{array}{c} Prob\left(\;C_{i}\;|\;x\;\right)=\frac{1}{1+exp\left(Af+B\right)}. \end{array}$$

In the testing section, a simplified version of the pairwise coupling strategy as proposed by Hastie and Tibshirani is used to combine the probabilistic outputs of all the APLSC classifiers as well as the output estimates of the posterior probabilities for all candidate classes. The combination equation is illustrated in

(2) $$\begin{array}{c} \tilde{p_{i}}=2\underset{j}{\sum }\frac{r_{ij}}{k\left(k-1\right)}. \end{array}$$

#### 4.2.4. Our Framework of TCM Data Mining

**Figure 6:**
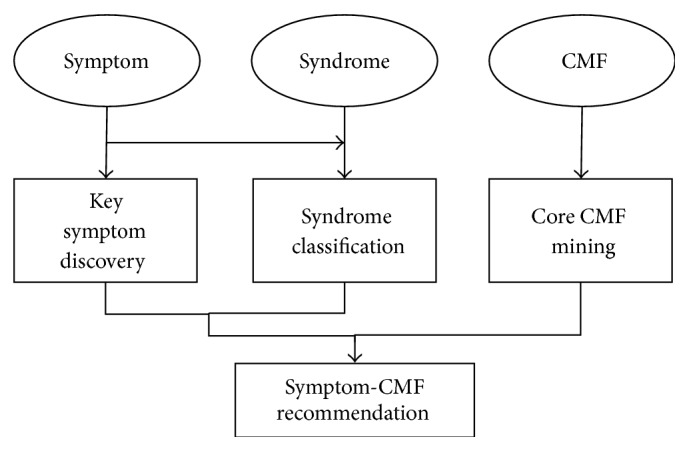
Framework of TCM clinical data analysis.

In TCM, hierarchical data analysis demands have risen.  Figure 6 demonstrates our framework analysis. The top layer includes items from clinical cases. Symptom, syndrome, and CMF are the three most important clinical items in which researchers are typically involved. The middle layer and bottom layer consist of four analysis directions that attract great attention in the field of TCM.

*(i) Key Symptom Discovery*. The Key Symptom Discovery is used to find the remarkable symptoms, which may be biomarkers for a specific disease or syndrome. As mentioned previously, items in templates are the summary of doctors' clinical knowledge. However, in the previous process, items are selected based on doctor's personal understanding and experience, which tends to be subjective and one-sided. Key Symptom Discovery discovers significant symptoms with objective algorithms, whereas the Feature Selection Group is usually used. 

*(ii) Syndrome Classification*. ISMAC helps doctors to diagnose disease/syndrome using the input symptoms and classification models. This speeds up knowledge dissemination in TCM by offering students more chances to study the clinical thoughts of the TCM veteran, as compared with traditional apprenticeship. Furthermore, ISMAC with a syndrome classification function may be deployed in outlying areas, helping junior doctors to improve diagnostic skills and treatment effects. 

*(iii) Core CMF Mining*. Core CMF is the basic Chinese Medicine Formula for a specific disease or syndrome. It is worthy of discovery since it reflects doctoral thought in clinical practice and the core of clinical knowledge. CMFs are also usually a valuable source of patent medicine. Mining CMFs could also bring great commercial value. Several methods embedded in ISMAC may be adopted to the core CMF mining. Algorithms in the Association Rule Group help find the closely associated herb subsets which are the candidates of core CMF. Clustering methods may be attempted to find herb clusters for CMF analysis. 

*(iv) Symptom-CMF Recommendation*. Symptom-CMF Recommendation is the combination and improvement of all three analyses in the middle layer. After a new patient inputs his symptoms, the corresponding classification model is called to judge the syndrome. The Key Symptom Discovery is embedded into the syndrome prediction operation for precision and speed. Core CMF for that syndrome can be recommended roughly to the patient. The customized treatment theory in TCM indicates that the CMF should either continue or remove some of the herbs, according to special symptoms of the patient. Thus, the Symptom-CMF Recommendation needs more models to learn the relationship between symptom difference and CMF modification. 

## 5. Example

After three years of observation of 11 TCM veteran practitioners, more than three thousand clinical records have been collected in ISMAC. Here, we take clinical data of fatty liver from Mr. Zhang Yunpeng, a known TCM liver specialist, as an example of demonstrating how to use ISMAC to manage and analyse clinical cases.

### 5.1. Template Design

The first step is to design the template. Usually, a template is designed by a specific TCM doctor and an expert team, as they are experienced in TCM diagnosis. Here, the itemset for a fatty liver template is supplied by Mr. Zhang's case research team, which has been conducting years of research on Mr. Zhang's clinical cases. The contents of the itemset are illustrated in Tables 1, 2, and 3.

### 5.2. Template Creation

With the summarized symptoms, syndromes, and therapeutic principles mentioned above, the corresponding IMA now can set up the template of Mr. Zhang for fatty Liver as the following steps.

**Step 1 .**

Select the entry* Inquiry and Diagnosis Template Maintenance* in the menu on the left side to go to its main page. Click the button* Add* to pop up the New Template Dialog.

**Step 2 .**

Assign the template a name; here* Fatty Liver* is entered. The serial number will be generated automatically.

**Step 3 .**

Input the name of TCM diseases or syndromes associated with this template. Notice that standard and expert-standard TCM disease have different ways of entry. Here in Mr. Zhang's template for fatty liver, expert-standard TCM diseases and syndromes are used, which are listed in Tables 1(a) and 2(a), respectively.

- Standard TCM diseases or syndromes: check the option* Use standard TCM disease*. Click the right button below to pop up the* Choose TCM Disease Dialog*. Select a disease from the* Disease Tree*.
- Expert-standard TCM diseases or syndromes: uncheck the option* Use standard TCM disease*. Two new text input boxes will replace the original text box for the manual entry of the TCM disease or respective syndrome.

**Step 4 .**

Input the therapeutic principles of this template. The ways of entering standard and expert-standard therapeutic principle are also different. For this template, we enter expert-standard therapeutic methods as shown in Table 2(b).

- Standard therapeutic principles: check the option* Use Standard Therapeutic Principle*. Select an item from the popup* Choose Therapeutic Principle Dialog*.
- Expert-standard therapeutic methods: uncheck the option* Use Standard Therapeutic Principle* and input the therapeutic principles manually.

**Step 5 .**

Input the standard or expert-standard WM diseases that this template deals with. The different ways of entering standard and expert-standard WM diseases are similar to those of the therapeutic principles mentioned above. For the template of fatty liver, we input the expert-standard WM diseases, which are shown in Table 1(b).

**Step 6 .**

Select the default dosage forms and default route of administration. The dosage forms might be injection, enema, decoction, and so forth. The administration routes could be “intravenous injection,” “enema,” “swallow,” and so forth. As summarized by the clinical experience of Mr. Zhang, decoction and swallow are chosen as default forms and are routed, respectively.

**Step 7 .**

Select the* First-visit Symptoms*, and* Examination Items*. The operation process is firstly, click the button* First-visit Symptoms*,* Return-visit Symptoms*, or* Examination Items* to switch the mode for data entry. Secondly, click the button* Add* to pop up the item selection dialog. Finally, check the needed items in the popup dialog. Here, for the template of fatty liver, the items listed in Tables 3(a) and 3(b) are added to both first-visit and return-visit tables, while there are no items for the examination table.

**Step 8 .**

Submit the form. A new template is created.

### 5.3. Data Collection and Analysis

Using the template created above, we have collected 63 clinical records for fatty liver. In the following, we will take syndrome classification and core CMF mining on the fatty liver data as an example of demonstrating how to use ISMAC for clinical case intelligent analysis.

#### 5.3.1. Syndrome Classification of Fatty Liver Data

After years of research, we have discovered that the TCM clinical diagnoses have specific characteristics like multiple possible syndromes, the complex classification of diseases, imbalanced data distribution, and small sample size, which make the classification of TCM syndrome based on symptoms quite challenging. In response to these problems, we use the MAPLSC algorithm: a pattern classification algorithm proposed to settle the imbalanced multiclass problem, and build a model on fatty liver data and compare its performance with other state-of-the-art algorithms.

*(a) Experiment Procedure*. First, target dataset needs to be retrieved. In this task, all 63 cases are involved, but not all attributions are required. We want a model that can help us classify patients' syndromes through the analysis of symptoms and examinations. So in this target dataset, 32 items listed in Tables 3(a) and 3(b) act as feature attributions. Each record in the target dataset has one class label that indicates which syndrome the patient suffers. The type of syndrome can be one listed in Table 2(a).

Next, a comparative approach should be carefully designed. Our object is to check whether MAPLSC outperforms other state-of-the-art methods, as it analyzes imbalance and multiclass problems. In this case, the first question is which methods it should be compared with. After careful consideration, APLSC PWC, APLSC Vote, SVM Prob, SVM Vote, and J48 are chosen. APLSC PWC and APLSC Vote are used to check whether our method of extending the APLSC algorithm to a multiclass domain is the best one. SVM related multiclass algorithms are chosen, because SVM is very powerful in building a classification model which does not consider data imbalance. Therefore, comparisons between MAPLSC and others can demonstrate whether data imbalance should be taken into consideration when building a classification model. J48 is also a powerful and general classification method which can be included. The second question regards which criterion should be exploited in the experiment, including microaverage accuracy, macroaverage, and macro-*F*1-measure. More details about these algorithms and criterions can be referred to in the original paper [25].

Finally, the learning task is set up. All the six algorithms mentioned above are added in the Algorithm Maintenance module with specific parameters. And then these algorithms, the three criterions, and the target dataset are collected in a New Learning Task Dialog to create the target learning task. Cross-validation is also configured to report more convincing results.

*(b) Experiment Results and Analysis*.  Table 4 illustrates the comparison results, from which we can observe that

(1) APLSC_PWC and APLSC_Vote are worse than MAPLSC on all the measurement rules.
(2) SVM related methods demonstrate poorer performance than APLSC related techniques on macro_average accuracy and macro_average *F*1-measure. However, higher micro_average accuracy values are recorded.
(3) J48 has no advantage on any metrics.

**Figure 7:**
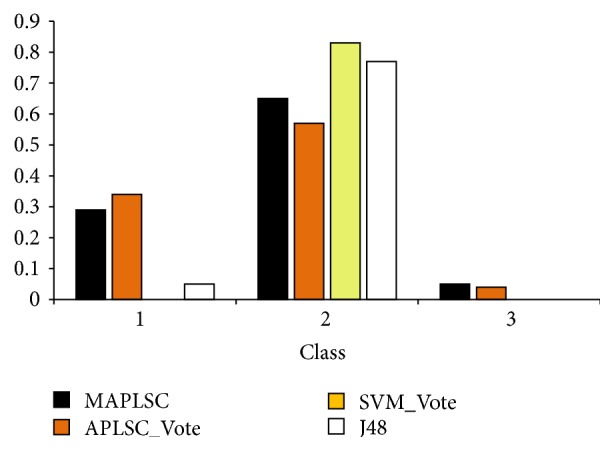
*F*1-measure of each class syndrome classification.

Observation (1) proves that our way of combining multiple classifiers is best. Observation (2) shows that MAPLSC is better with macro criterion, but worse with micro criterion. As macro criterion weighs equally all the classes while micro average weighs equally all the examples, MAPLSC can balance performance among classes. We draw Figure 7 to see performance distributions of different methods and different classes. In Figure 7, MAPLSC successfully classifies some examples of the difficult class 3. However, SVM Vote and J48 cannot identify any examples, and the *F*1-Measure diverges greatly among classes. This verifies that the MAPLSC has the ability to balance performance among classes. In the task of syndrome classification, MAPLSC performs well on almost all classes. And for some difficult class, the MAPLAC can also work.

#### 5.3.2. Core Formula Mining on Fatty Liver Clinical Records

Referring to the TCM theory of Mr. Zhang, a core CMF for fatty liver therapy exists. From Section 4.2.3, we know that core CMF mining is very valuable. Occurrence frequency computing and Apriori are two popular algorithms for finding frequent patterns. The experimental procedure is similar to that of syndrome classification. Here, we omit the description of experimental procedure and elaborate the experiment results and analysis.

*(a) Experiment Result and Analysis*

**Figure 8:**
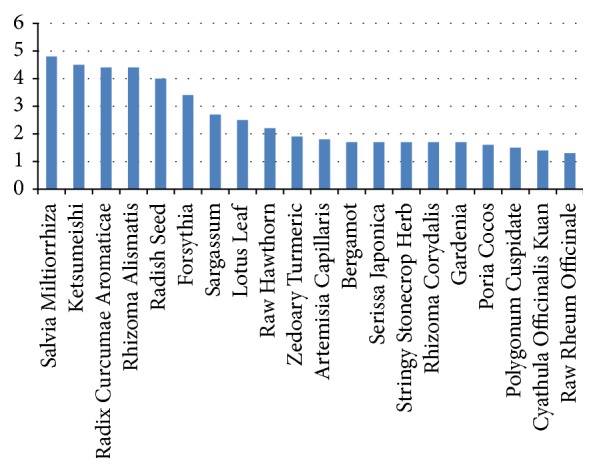
The first 20 most frequently used herbs and their normalized frequency.

**Figure 9:**
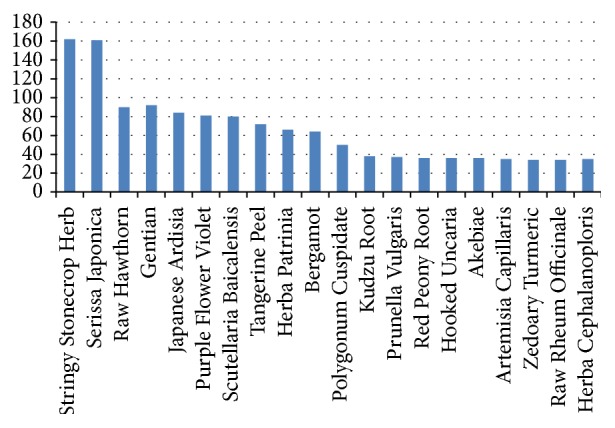
The first 20 most frequently used herbs and their normalized frequency except 8 herbs in basic core formula.

- Core formula for fatty liver: using the* occurrence frequency computing* method and other visualization functions provided by ISMAC, the first 20 herbs most frequently used in the CMFs for fatty liver can be found in Figure 8. The first 8 herbs should be the basic core formula for fatty liver, which are* salvia miltiorrhiza*,* ketsumeishi*,* radix curcumae aromaticae*,* rhizoma alismatis*,* radish seed*,* forsythia*,* sargassum*, and* lotus leaf*.
- Core formula for syndrome of intermingled phlegm and blood stasis and retained dampness heat toxin: dataset in this task contains only clinical records belonging to the syndrome of intermingled phlegm and blood stasis and retained dampness heat toxin. By removing the 8 herbs belonging to the basic core formula, there will be 137 herbs used for this syndrome. Once again,* occurrence frequency computing* is called to list the first 20 frequent herbs among 137 herbs. The result is illustrated in Figure 9, which shows us that the occurrence of the third herb* born hawthorn* goes down obviously compared with the first two herbs* stringy stonecrop* and* serissa japonica*. Thus, these two herbs are considered to be additional herbs of core formula for this syndrome.
- Employing* Apriori* to find frequent patterns in this dataset, we get the results in Table 5. Agreeing with the result using* occurrence frequency computing*, the most frequent 3-itemset is far less than the most frequent 2-itemset as indicated in that table.
- Synthetically taking results from these two methods into account, we draw a conclusion that* stringy stonecrop* and* serissa japonica*, together with the 8 herbs in basic core formula, constitute the core formula for the syndrome of intermingled phlegm and blood stasis and the retained dampness of heat toxin.
- Core formula for syndrome of intermingled phlegm and blood stasis: with the same analysis method and procedure mentioned above, we find that the core formula for the syndrome of intermingled phlegm and blood stasis is* hawthorn*,* aloe*, and* snakegourd seed*.
- Core formula for syndrome of intermingled phlegm and blood stasis and ascendant hyperactivity of liver yang: using the same way of analysis, the core formula for the syndrome of intermingled phlegm and blood stasis and ascendant hyperactivity of liver yang is* patrinia*,* rhixoma gastrodiae*,* stringy stonecrop herb*, and* serissa japonica*.

## 6. Conclusion

The paper proposes an intelligent ISMAC System for TCM clinical case management and analysis. Customized Templates for data entry may be designed exactly according to the clinical diagnosis and treatment characteristics of a certain physician. State-of-the-art data analysis and mining methods have been integrated and improved to discover hidden knowledge. ISMAC is dedicated to TCM clinical case archiving, TCM theory verification, latent knowledge discovering, young doctor tutoring, and so forth. Having cooperated with many TCM veteran practitioners, ISMAC has collected more than three thousand clinical records, for which a great deal of clinical records mining has been conducted. And some of the data analysis results are verified in clinical examination. In the future, more clinical cases are necessary, and the customized management function of ISMAC will be improved by considering more clinical requirements and real-life problems. More data analysis methods should be experimented with for TCM clinical cases. For example, more than one syndrome would be tagged to a clinical diagnosis, indicating that syndrome prediction is a multilabel problem. We preliminarily attempted multilabel *k* nearest neighbor algorithm to analyze the syndrome classification of TCM coronary heart disease dataset [13], but more investigation is needed. What is more, thousands of symptoms may be carried in TCM clinical examination, symptoms contributing mostly to syndrome diagnosis are of vital concern. Selecting appropriate symptoms for clinical diagnosis may be modeled as a multilabel feature selection problem in machine learning. HOML may succeed in the selection of relevant symptoms by using wrapper methods. It is a time-consuming technique, and symptom reduction remains a challenging topic.

## Figures and Tables

**Table:** (a) TCM diseases

Stuffiness of liver	
Vertigo	

**Table:** (b) WM diseases

Fatty liver	
Hyperlipaemia	
Hypertension	

**Table:** (a) TCM syndromes

Syndrome class number	

Intermingled phlegm and blood stasis and retained dampness of heat toxin	

Intermingled phlegm and blood stasis	

Intermingled phlegm and blood stasis and ascendant hyperactivity of liver yang	

**Table:** (b) TCM therapeutic principle

Blood-activating and stasis-dissolving, dispersing stagnated liver qi and removing obstruction in the channels	

Blood-activating and stasis-dissolving, clearing away heat and toxic materials	

Blood-activating and stasis-dissolving, clearing toxic materials and resolving hard lump	

Blood-activating and stasis-dissolving, calming Liver to stop endogenous wind	

Blood-activating and stasis-dissolving, nourishing the heart and restoring the pulse	

**Table:** (a) Main symptoms

Whether in first-visit	
Tired or not	
State of tongue	
State of tongue coat	
State of tongue proper	
Pulse condition	
Trigger of hypochondriac pain	
Type of hypochondriac pain (left side)	
Type of hypochondriac pain (right side)	
Trigger of liver pain	
Type of liver pain	
Level of liver pain	

**Table:** (b) Estabilished symptoms

Nausea	
Loose stool	
Constipation	
Sou huang	
Backaches	
Hline nai cha	
Lusterless in face	
Uncomfortable in epigastrium	
Thirsty	
Acid regurgitation	
Yellow eyes	
Abdominal distention	
Depression	
Sallow complexion	
Nausea	
Belch	
Vomit	
Edema of lower extremity	
Bitter taste of mouth	
Restless sleep at night	

**Table 4:** Comparative results on syndrome classification.

Metrics	APLSC_Vote	MAPLSC	APLSC_PWC	SVM_Vote	SVM_Prob	SVM_PWC	J48
Macro avg_acc	38.33 ± 6.81	40.07 ± 6.69	38.74 ± 7.22	33.33 ± 0.00	32.79 ± 0.32	32.79 ± 0.32	30.11 ± 2.06
Micro avg_acc	45.78 ± 5.00	52.42 ± 5.49	51.8 ± 5.42	71.88 ± 0.00	70.70 ± 0.69	70.70 ± 0.69	62.73 ± 3.49
Macro avg_f1meas	34.73 ± 5.19	38.14 ± 5.57	37.13 ± 5.72	27.88 ± 0.00	27.61 ± 0.16	27.61 ± 0.16	27.36 ± 2.27

**Table 5:** The most frequent *n*-itemsets and their frequency.

Number of items	Frequency	The most frequent *n*-itemset
1	162	Stringy Stonecrop Herb

2	158	Stringy Stonecrop Herb, Serissa Japonica

3	89	Stringy Stonecrop Herb, Serissa Japonica, Gentian

4	56	Stringy Stonecrop Herb, Serissa Japonica, Gentian, Japanese Ardisia

5	37	Stringy Stonecrop Herb, Serissa Japonica, Hawthorn, Gentian, Japanese Ardisia

6	25	Stringy Stonecrop Herb, Serissa Japonica, Hawthorn, Gentian, Tangerine Peel, Japanese Ardisia

## References

[1] Zhu C. (2008). Chen Zhu interview. China's modern medical minister. Interview by Richard Stone. *Science (New York, N.Y.)*.

[2] Tang J.-L., Liu B.-Y., Ma K.-W. (2008). Traditional Chinese medicine. *The Lancet*.

[3] Anonymous (2003). *The Inner Canon of Emperor Huang*.

[4] Chinese National Bureau of Statistics (2008). The 2008 National Social Statistical Data of China. http://www.stats.gov.cn/tjsj/ndsj/2009/indexch.htm.

[5] Konkimalla V. B., Efferth T. (2008). Evidence-based Chinese medicine for cancer therapy. *Journal of Ethnopharmacology*.

[6] Rao J. K., Mihaliak K., Kroenke K., Bradley J., Tierney W. M., Weinberger M. (1999). Use of complementary therapies for arthritis among patients of rheumatologists. *Annals of Internal Medicine*.

[7] Wang L., Zhou G.-B., Liu P. (2008). Dissection of mechanisms of Chinese medicinal formula Realgar-Indigo naturalis as an effective treatment for promyelocytic leukemia. *Proceedings of the National Academy of Sciences of the United States of America*.

[8] Chang T.-T., Sun M.-F., Chen H.-Y. (2011). Screening from the world's largest TCM database against H1N1 virus. *Journal of Biomolecular Structure & Dynamics*.

[9] Diener H.-C., Kronfeld K., Boewing G. (2006). Efficacy of acupuncture for the prophylaxis of migraine: a multicentre randomised controlled clinical trial. *Lancet Neurology*.

[10] Zhou X., Chen S., Liu B. (2010). Development of traditional Chinese medicine clinical data warehouse for medical knowledge discovery and decision support. *Artificial Intelligence in Medicine*.

[11] Zhou X., Peng Y., Liu B. (2010). Text mining for traditional Chinese medical knowledge discovery: a survey. *Journal of Biomedical Informatics*.

[12] Poon S. K., Poon J., McGrane M. (2011). A novel approach in discovering significant interactions from TCM patient prescription data. *International Journal of Data Mining and Bioinformatics*.

[13] Liu G.-P., Li G.-Z., Wang Y.-L., Wang Y.-Q. (2010). Modelling of inquiry diagnosis for coronary heart disease in traditional Chinese medicine by using multi-label learning. *BMC Complementary and Alternative Medicine*.

[14] Tian J., Xue J., Dai Y., Chen J., Zheng J. (2008). A novel software platform for medical image processing and analyzing. *IEEE Transactions on Information Technology in Biomedicine*.

[15] Masseroli M., Marchente M. (2008). X-PAT: a multiplatform patient referral data management system for small healthcare institution requirements. *IEEE Transactions on Information Technology in Biomedicine*.

[16] Ku H.-H., Huang C.-M. (2010). Web2OHS: a Web2.0-based omnibearing homecare system. *IEEE Transactions on Information Technology in Biomedicine*.

[17] Chen H., Wu Z., Huang C. (2003). TCM-Grid: weaving a medical grid for traditional Chinese medicine. *Computational Science—ICCS 2003*.

[18] Zhou X., Wu Z., Lu W. TCMMDB: a distributed multidatabase query system and its key technique implemention.

[19] You M., Li G. Z., Zeng X. Q. (2008). A personalized traditional Chinese medicine system in the case of Cai's gynecology. *International Journal of Functional Informatics and Personalised Medicine*.

[20] You M., Yan S.-X., Li G.-Z. Customized management of clinical data in traditional Chinese medicine.

[21] Hall M., Frank E., Holmes G., Pfahringer B., Reutemann P., Witten I. H. (2009). The WEKA data mining software: an update. *ACM SIGKDD Explorations Newsletter*.

[22] Agrawal R., Srikant R. Fast algorithms for mining association rules.

[23] You M., Chen Y., Li G. (2010). Im-IG: a novel feature selection method for imbalanced problems. *Journal of Shangdong University (Engineering Science)*.

[24] Qu H.-N., Li G.-Z., Xu W.-S. (2010). An asymmetric classifier based on partial least squares. *Pattern Recognition*.

[25] You M., Zhao R. W., Li G. Z., Hu X. (2011). MAPLSC: a novel multi-class classifier for medical diagnosis. *International Journal of Data Mining and Bioinformatics*.

